# Phosphorylation of AIB1 at Mitosis Is Regulated by CDK1/CYCLIN B

**DOI:** 10.1371/journal.pone.0028602

**Published:** 2011-12-07

**Authors:** Macarena Ferrero, Juan Ferragud, Leonardo Orlando, Luz Valero, Manuel Sánchez del Pino, Rosa Farràs, Jaime Font de Mora

**Affiliations:** 1 Laboratory of Cellular and Molecular Biology, Centro de Investigación Príncipe Felipe (CIPF), Valencia, Spain; 2 Laboratory of Proteomics, CIPF, Valencia, Spain; 3 Laboratory of Cytomics, CIPF, Valencia, Spain; University of Minnesota, United States of America

## Abstract

**Background:**

Although the AIB1 oncogene has an important role during the early phase of the cell cycle as a coactivator of E2F1, little is known about its function during mitosis.

**Methodology/Principal Findings:**

Mitotic cells isolated by nocodazole treatment as well as by shake-off revealed a post-translational modification occurring in AIB1 specifically during mitosis. This modification was sensitive to the treatment with phosphatase, suggesting its modification by phosphorylation. Using specific inhibitors and in vitro kinase assays we demonstrate that AIB1 is phosphorylated on Ser728 and Ser867 by Cdk1/cyclin B at the onset of mitosis and remains phosphorylated until exit from M phase. Differences in the sensitivity to phosphatase inhibitors suggest that PP1 mediates dephosphorylation of AIB1 at the end of mitosis. The phosphorylation of AIB1 during mitosis was not associated with ubiquitylation or degradation, as confirmed by western blotting and flow cytometry analysis. In addition, luciferase reporter assays showed that this phosphorylation did not alter the transcriptional properties of AIB1. Importantly, fluorescence microscopy and sub-cellular fractionation showed that AIB1 phosphorylation correlated with the exclusion from the condensed chromatin, thus preventing access to the promoters of AIB1-dependent genes. Phospho-specific antibodies developed against Ser728 further demonstrated the presence of phosphorylated AIB1 only in mitotic cells where it was localized preferentially in the periphery of the cell.

**Conclusions:**

Collectively, our results describe a new mechanism for the regulation of AIB1 during mitosis, whereby phosphorylation of AIB1 by Cdk1 correlates with the subcellular redistribution of AIB1 from a chromatin-associated state in interphase to a more peripheral localization during mitosis. At the exit of mitosis, AIB1 is dephosphorylated, presumably by PP1. This exclusion from chromatin during mitosis may represent a mechanism for governing the transcriptional activity of AIB1.

## Introduction

The overexpression of AIB1, a transcriptional coactivator, promotes pre-neoplastic changes and cancer initiation in animal models [1], [2]. The mechanisms by which AIB1 alter cell growth involve a variety of signaling pathways including ER, IGF/PI3K/AKT, HER2, NF-κB and Ets (reviewed in [3]. Interestingly, overexpression of AIB1 is correlated with tumor invasiveness and high levels of Twist [4]. AIB1 also functions as a coactivator of AP-1 to up-regulate the expression of MMP-7 and MMP-10 in breast cancer cell lines [5]. However, the expression level of AIB1 is not the only determinant of its oncogenic potential since post-translational modifications such as phosphorylation, ubiquitylation, sumoylation, and acetylation (reviewed in [6] have been demonstrated to modulate the activity of AIB1. Moreover, the sub-cellular localization of AIB1 is an important parameter in the regulation of this coactivator [7].

Most of the studies related to AIB1 and cell-cycle regulation have focused on G1/S progression. During this phase, AIB1 is localized, along with ERα, to the active promoter of cyclin D1 [8]. In addition, AIB1 promotes G1 progression by coactivating the transcription of E2F1 [9]. Cyclin A and cyclin E are regulated transcriptionally by the complex AIB1/E2F1 in the G1 to S transition [10]. AIB1 also appears to have an important role during or after S phase of the cell cycle [1], [10]. However, very little is known about the function of AIB1 during mitosis. One study has shown that AIB1 is essential for the phosphorylation of histone H3 at serine 10 [11], a conserved molecular mechanism which is accompanied by chromosome condensation in mitosis [12].

The events of mitosis, including reorganization of the cellular architecture and chromosome condensation, require fine tuning to ensure the precision of the cell-cycle. These processes are coordinated by different families of kinases that trigger protein phosphorylation cascades. The cyclin-dependent kinase Cdk1 is a key regulator of the onset of mitosis. Activation of Cdk1 during late G2 initiates the cellular reorganization which also involves the activity of three other kinase families: Aurora, Polo-like (Plk) and NIMA-related (Nrk) kinases. Although both Aurora A and Plk1 are activated during early G2 [13], their activity as well as their expression levels peak during M phase [14], [15], as part of a feedback loop with Cdk1. Aurora A and Plk1 are not required for onset of mitosis, revealing a unique role of Cdk1 in G2/M progression [16]. Activation of Plk1 by Aurora A facilitates mitotic entry as well as checkpoint recovery [13], [17]. In addition, Nrk kinases participate in microtubule dynamics from late G2 through mitosis [18].

With the present study, we demonstrate that AIB1 associates with Cdk1 and undergoes phosphorylation just at the entry to mitosis. This phosphorylation event is dependent on cyclin B but not cyclin A, further supporting the phosphorylation of AIB1 at the onset of mitosis. Interestingly, neither ubiquitylation nor protein degradation were associated with this phosphorylation. AIB1 remains phosphorylated throughout M phase and is dephosphorylated only at the exit from mitosis, coinciding with activation of PP1 and the inactivation of Cdk1. Importantly, phosphorylation at the defined sites did not alter the transcriptional activity of AIB1 but rather excludes this factor from chromatin during mitosis, perhaps reflecting a mechanism to prevent abnormal transcription during M phase. Collectively, these results reveal a novel mechanism for regulating AIB1 during mitosis by which chromatin exclusion and subcellular redistribution of AIB1 during mitosis may restrict its transcriptional activity.

## Results

### The electrophoretic mobility of AIB1 is reduced in mitosis

**Figure 1:**
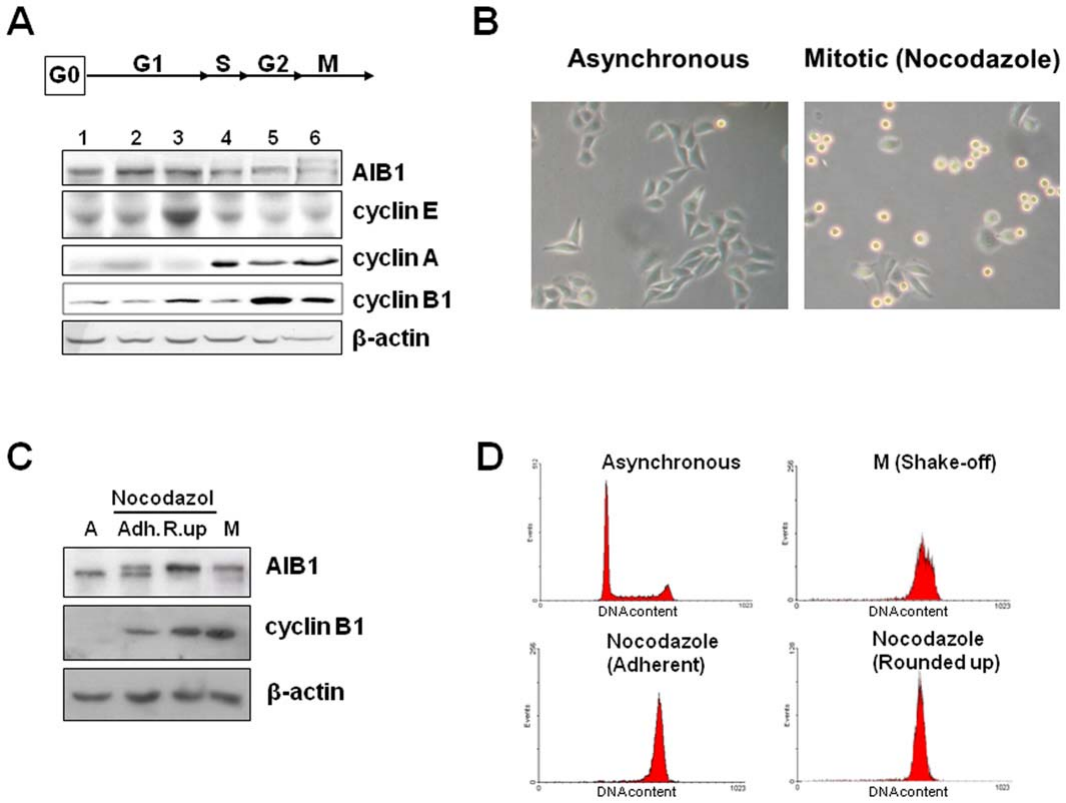
Post-translational modification of AIB1 occurs at the entry to mitosis. (A) Western blot analysis of HeLa cells arrested at the different stages of the cell-cycle as indicated in the scheme. Anti-AIB1 antibodies reveal an electrophoretic mobility shift in mitotic cells (lane 6). Antibodies against indicated cyclins were used to confirm cell-cycle progression through the different stages. β-actin was used as loading control. Chemically-induced cell cycle arrest was produced using the following drugs at the doses and times described in materials and methods: lane 1: cyclosporin A; lane 2: wortmannin; lane 3: L-mimosine; lane 4: hydroxyurea; lane 5: etoposide, and lane 6: nocodazole. (B) Morphology of asynchronous culture of HeLa cells and cells treated with nocodazole to arrest them at the beginning of mitosis. Rounded up cells (light refracting) are cells that have entered mitosis. The increased number of rounded uping cells under nocodazole treatment visually confirms the enriched mitotic population. (C) Western blot analysis of asynchronous growing cells (A), nocodazole-treated cells and mitotic cells (M) isolated from an asynchronous culture by shake-off. Nocodazole-treated cells were divided into rounded up cells (R.up) and those that still remained attached (Adh.) to the surface of the culture dish. (D) Flow cytometry analysis was used to confirm the DNA content of cells treated as indicated in (C).

Regulation of AIB1 activity and stability is mediated by several post-translational modifications. To study the cell cycle-dependent post-translational modifications of AIB1, we analyzed synchronized cultures of HeLa cells. These cells were arrested at different stages of the cell cycle (see scheme above the western blots in Figure 1A) and subsequently, analyzed by Western blotting. When cells were arrested by nocodazole in prometaphase, AIB1 was detected as a doublet (Figure 1A, lane 6). Similar results were obtained from other tumor cell lines arrested with nocodazole (data not shown), suggesting that this modification of AIB1 during cell-cycle arrest is not specific to HeLa cells.

Nocodazole-treated cells round-up and detach from the culture plates (Figure 1B), a morphology typical of mitosis. We used this fact to separate fully mitotic cells (rounded up cells) from those that had not yet entered mitosis (adherent cells). Interestingly, the only band detected by anti-AIB1 antibodies in rounded up cells was the upper band (slower mobility) of the doublet observed previously in nocodazole-arrested cultures (Figure 1C). Consistent with this, the slower migrating band was the only one detected in mitotic cells selected by shake-off from an asynchronous culture (a classical method for isolating mitotic cells by mechanical agitation), suggesting that it was not an artifact of the nocodazole treatment (Figure 1C, lane M). In parallel, flow cytometry analysis confirmed that the DNA content of nocodazole-treated and mitotic shake-off cells was double that of control cells, as expected for cells at G2/M (Figure 1D). These results demonstrate that AIB1 undergoes post-translational modification at mitosis that significantly retards its electrophoretic mobility.

### AIB1 is phosphorylated during mitosis

**Figure 2:**
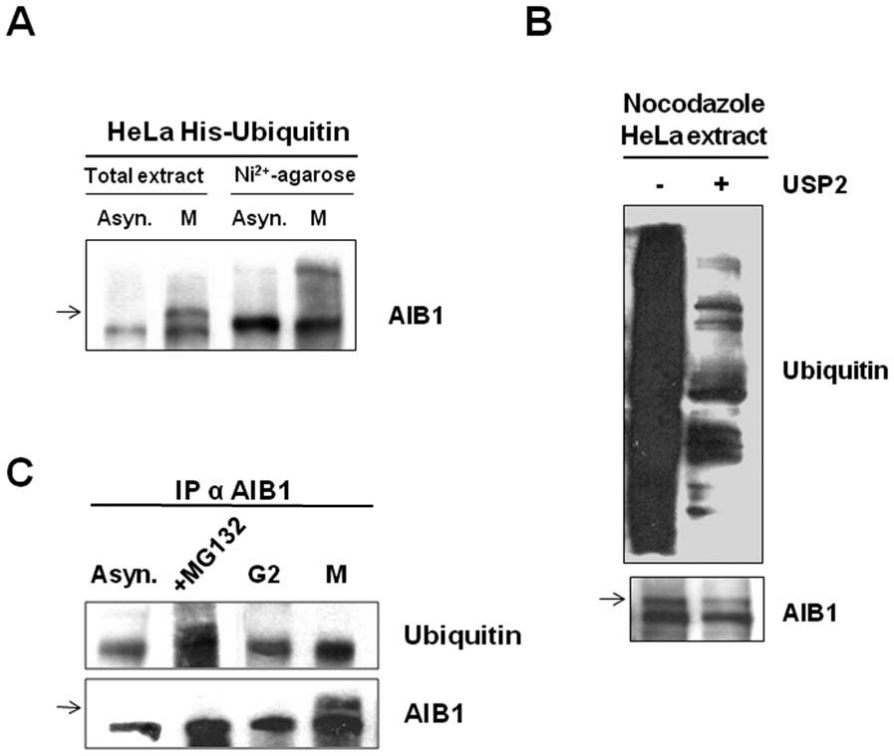
Post-translational modification of AIB1 at mitosis does not correlate with ubiquitination. (A) HeLa cells constitutively expressing 6xhistidine tagged-ubiquitin were grown asynchronously or arrested at mitosis with nocodazole and cell lysates were either directly analyzed (total extract) or subjected to purification through a Ni^2+^-agarose column and further analyzed by western blotting. Arrow indicates the AIB1 mitotic-specific band. (B) MCF-7 cell extracts (30 µg, 1∶100) from arrested cells with nocodazole were subjected to enzymatic assay with the 300 ng of deubiquitinating enzyme GST-USP2 in 50 µl deubiquitylation buffer (50 mM Tris-HCl pH 7.5, 1 mM EDTA, 1 mM DTT). Samples were analyzed by western blot with anti-mono, poly-ubiquitin antibodies and anti-AIB1 antibodies. The altered electrophoretic mobility of the AIB1 band is indicated with an arrow, demonstrating insensitivity to USP2 activity. (C) HeLa cells were grown asynchronously, or treated with the proteasome inhibitor MG132, or with etoposide to arrest them at G2 or with nocodazole to arrest them at mitosis (M). Cell lysates were immunoprecipitated with AIB1 antibodies and immunocomplexes were analyzed by western blotting with anti-mono, poly-ubiquitin antibodies and anti-AIB1 antibodies. The arrow indicates the mitosis-specific AIB1 band that is not detected with ubiquitin antibodies.

We and others have shown previously that AIB1 is ubiquitilated and degraded by the proteasome [7], [19], [20]. To identify the AIB1 modification which occurs at or during mitosis, we first assessed ubiquitilation. The HeLa cell line constitutively expressing 6xhistidine-tagged ubiquitin constitutes a very appropriate biological system for this study. Cells can be lysed under denaturing conditions and ubiquitilated proteins bind to Ni^2+^-column without undergoing degradation or ubiquitylation during the process. Ubiquitilated AIB1 was enriched in the Ni^2+^-bound fraction of asynchronous cells and displayed a slight retardation of electrophoretic mobility in contrast to the total extract (Figure 2A). The ubiquitin-enriched fraction from mitotic cells revealed that AIB1 was also ubiquitilated (right M lane, Figure 2A). However, ubiquitilated AIB1 at mitosis did not display the same mobility as in whole cell extracts (compare both M lanes, Figure 2A). These results suggest that AIB1 is weakly ubiquitilated during mitosis but this does not produce the same band-shift as detected in whole cell lysates. We then performed an *in vitro* assay with USP2, a potent deubiquitilase. As expected, many proteins from the whole cell extract were deubiquitilated after USP2 treatment; however, AIB1 from mitotic cells was only slightly, if at all, altered by USP2 (Figure 2B). To further evaluate whether AIB1 is ubiquitilated during M phase, we used a third approach with antibodies that detect both, mono- and poly-ubiquitilated proteins. However, the results of the Western blots reveal that these antibodies did not recognize AIB1 from mitotic cells (Figure 2C, compare upper and lower panels). Collectively, these experiments exclude the ubiquitilation pathway as a potential explanation for the retarded electrophoretic mobility of AIB1 in cells at M phase.

**Figure 3:**
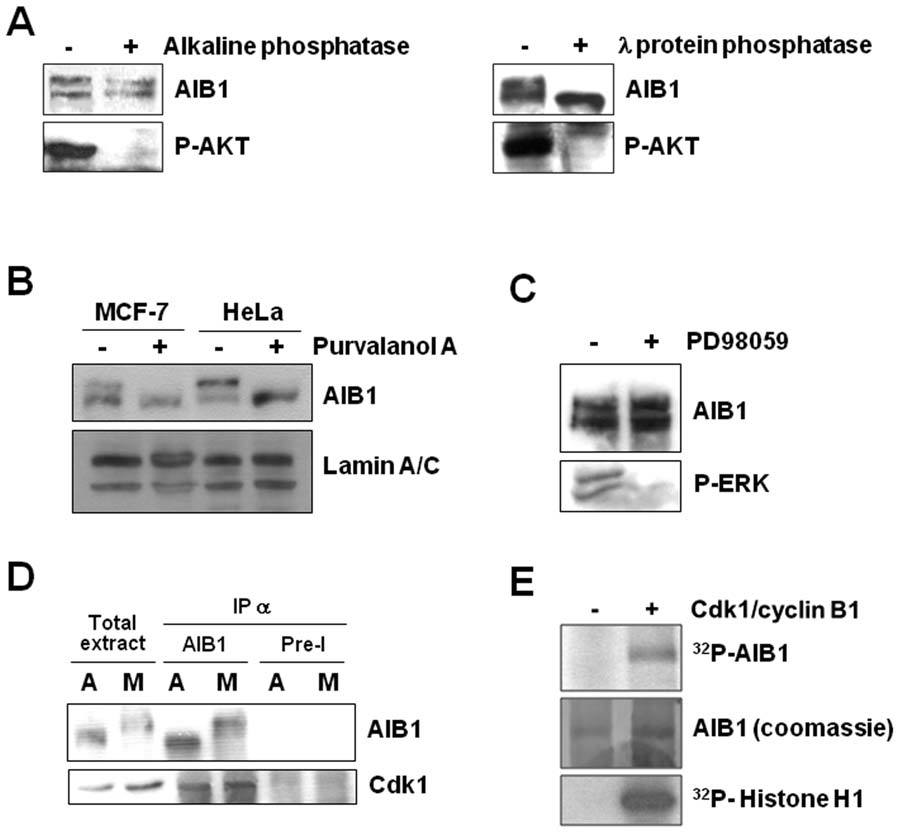
AIB1 is phosphorylated at entry to M phase by Cdk1/cyclin B. (A) Western blot analysis of 30 µg of cleared lysates from nocodazole-arrested HeLa cell which were treated with the indicated phosphatases for 1.5 h at 30°C. Phospho-Akt antibodies were used as a control of the enzymatic activity. (B) Cells arrested at mitosis with nocodazole were further treated with vehicle alone (−) or with the Cdk1 inhibitor purvalanol A 10 mM (+) during 3 hours. Anti-lamin A/C antibodies were used to control loading. (C) Mitotic cells were treated with the MEK inhibitor PD98059 20 µM (+) or with vehicle alone (−) and cell lysates were analyzed by western blotting with AIB1 antibodies. Phospho-ERK specific antibodies were used as control for the efficiency of the treatment. (D) Western blot analysis of immunoprecipitated complexes using anti-AIB1 antibodies or pre-immune serum (Pre-I) from lysates of asynchronous HeLa cells (A) or from cells arrested with nocodazole (M). (E) Human AIB1 was subcloned in pFASTBac HTa and baculovirus were produced to express the recombinant full-length His6-AIB1 in Sf9 cells using the system BacPak (Clontech). Recombinant protein was purified by standard Ni^2+^-NTA affinity chromatography followed by desalting with a molecular exclusion column. Purified AIB1 (middle panel) was incubated with [γ-^32^P]ATP in the presence (+) or absence (−) of recombinant Cdk1/cyclin B1 for 1 hour at 30°C (upper panel). Reactions were resolved by SDS-PAGE, the gel was dryed and exposed to an X-ray film. 1 µg of histone H1 (Sigma) was used as a positive control (bottom panel).

**Figure 4:**
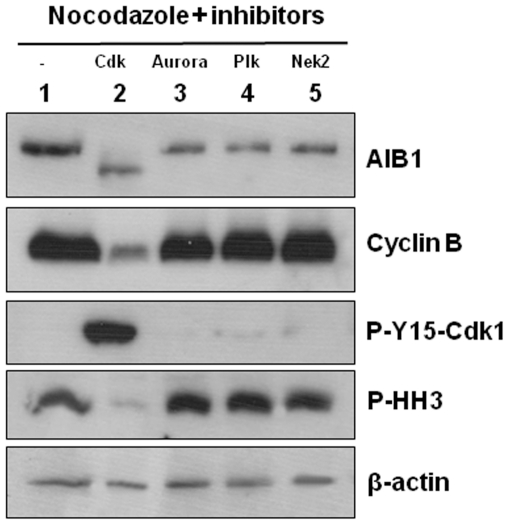
AIB1 is not a substrate of Aurora A, B, Plks or Nek2. Floating HeLa cells arrested at mitosis with nocodazole were further treated for three more hours with vehicle alone as control (lane 1), 10 µM purvalanol A (lane 2), 1.25 µM Aurora kinase inhibitor II (lane 3), 7 µM Plk inhibitor III (lane 4) or 100 µM of the Hec1/Nek2 inhibitor I (lane 5). Cells were lysed and analyzed by westernblotting against the indicated molecular species. Importantly, exposure of cells to the Cdk1 inhibitor caused the desphosphorylation of AIB1 together with a rapid mitotic exit (lane 2) to a similar extent as previously reported with other inhibitors [31], [32]. On the other hand, exposure of mitotic cells to inhibitors specific for Aurora A, B, Plks or Nek2 did not show any significant effect on AIB1 phosphorylation, suggesting that neither of them is a kinase for AIB1 under the experimental conditions tested. The protein kinase inhibitor staurosporine causes morphological changes at mitosis in HeLa cells, including the decondensation of chromosomes and the reformation of nuclear membrane [33]. Treatment with the different kinase inhibitors at the concentrations indicated by the supplier caused similar morphological changes plus the reattachment of cells to the surface of the petri dish (data not shown), suggesting that the inhibitors indeed exerted a biologically relevant activity.

We then explored whether the altered mobility of AIB1 detected in mitotic cells is the result of phosphorylation. Thus, lysates from cells arrested at mitosis were subjected to various protein phosphatases. Treatment with λ protein phosphatase, but not alkaline phosphatase, restored the AIB1 doublet to the faster migrating single band observed in asynchronized cultures (Figure 3A), suggesting that alterations of mobility observed in mitotic cells were due mostly to phosphorylation. To test whether Cdk1 might participate in the phosphorylation of AIB1 at M phase, we used the Cdk inhibitor purvalanol A. Cells arrested at mitosis with nocodazole and further treated with purvalanol A did not display the band shift characteristic of AIB1 at M phase (Figure 3B). Although highly specific for Cdk, purvalanol A has also been reported to weakly inhibit ERKs. Given that AIB1 can be phosphorylated by ERKs [21], nocodazole-arrested cells were subsequently treated with the MEK inhibitor PD98059 to specifically block the phosphorylation and activation of ERKs during mitosis. Although PD98059 inhibited ERKs phosphorylation, the electrophoretic mobility of AIB1 was not altered by this treatment (Figure 3C). Similarly, treatment of mitotic cells with specific inhibitors of Aurora A/B, Plk1-4 or Nek2 did not cause dephosphorylation of AIB1 (Figure 4). Thus, these results suggest that the phosphorylation of AIB1 at mitosis is Cdk1-dependent. To further test this hypothesis, we expressed full-length AIB1 in Sf9 cells and the purified protein was subjected to an *in vitro* kinase assay with active Cdk1/cyclin B1. AIB1 was detected as a radio-labeled protein (Figure 3E), demonstrating that it is a direct substrate of Cdk1/cyclin B1 *in vitro*. Consistent with this, immunoprecipitation of AIB1 from asynchronous (A) or mitotic (M) culture lysates revealed the presence of Cdk1 in AIB1 immunocomplexes, suggesting a functional relationship *in vivo* between these proteins (Figure 3D).

### AIB1 is dephosphorylated at the exit from mitosis

**Figure 5:**
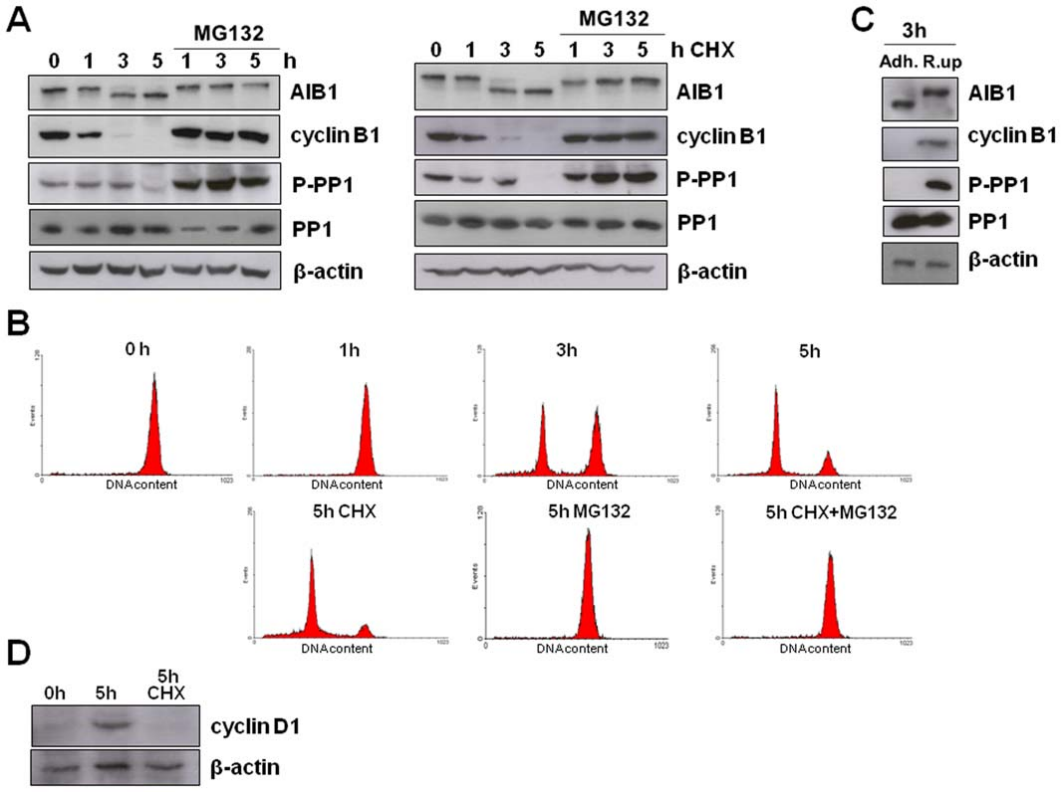
Desphosphorylation of AIB1 occurs at exit from mitosis. (A) Rounded up HeLa cells arrested at mitosis with nocodazole were washed with PBS and allowed to progress through mitosis for the times indicated, in the presence or absence of 20 µM MG132 and 10 µg/ml cycloheximide (CHX). Western blot analysis was performed for AIB1 to monitor its phosphorylation status. β-actin were assessed as loading control. (B) Flow cytometry analysis of HeLa cells treated as above. (C) HeLa cells arrested at mitosis were allowed to progress through cell cycle and at 3 hours, the remaining rounded up cells (R.up) were separated from adherent cells (Adh.) and analyzed by western blotting. (D) Rounded up HeLa cells recovered after nocodazole treatment were washed with PBS and allowed to progress through mitosis for the indicated times in the absence or presence of 10 µg/ml cycloheximide (CHX).

One consequence of the exit from mitosis is the dephosphorylation of multiple structural and regulatory proteins; the protein phosphatase 1 (PP1) is responsible for the majority of this dephosphorylation activity [22]. Cdk1 phosphorylates PP1 at threonine 320 and inhibits its activity [23]. To assess the phosphorylation status of AIB1 at the end of M phase, rounded up HeLa cells arrested at mitosis with nocodazole were isolated, washed and allowed to proceed through mitosis in normal growth medium. The majority of AIB1 was dephosphorylated 5 hours after release from the nocodazole blockade, coinciding with the degradation of cyclin B1 and the dephosphorylation (and therefore the reactivation) of PP1 (Figure 5A, left panels). In parallel, flow cytometry analysis confirmed that although some cells were reentering G1 after 3 hours, at 5 hours most of them had exited mitosis and were already at G1 (Figure 5B, upper graphs). In addition, treatment with the proteasome inhibitor MG132 arrested cells at mitosis (Figure 5B, lower graph). Western blot analysis of these lysates revealed that MG132 blocked cyclin B1 degradation, PP1 reactivation and the de-phosporylation of AIB1 at exit of mitosis (Figure 5A, right lanes in the left panels). These results suggest that dephosphorylation of AIB1 at the termination of M phase coincides with the re-activation of PP1.

To further substantiate the dephosphorylation of AIB1 at the exit from mitosis, rounded up cells were separated from adherent cells 3 hours after their release from nocodazole-mediated arrest and were analyzed by western blotting. Interestingly, AIB1 was completely dephosphorylated in adherent cells that represent G1, whereas the rounded up fraction of cells retained at M contained only phosphorylated AIB1 as evidenced by the slower mobility of this band (Figure 5C).

**Figure 6:**
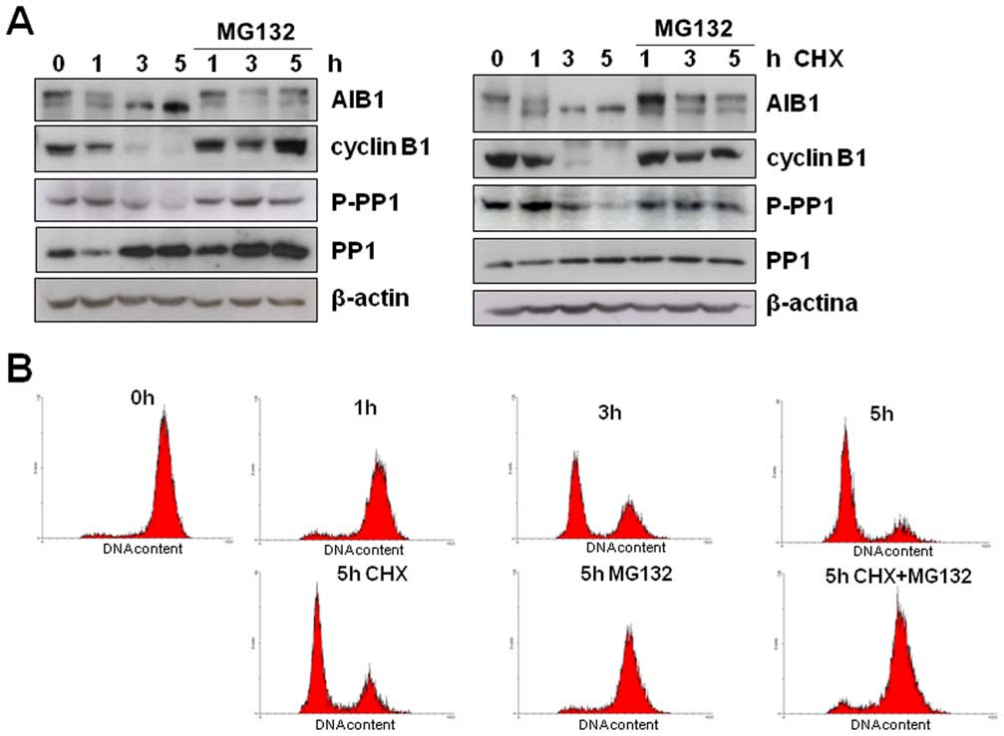
Desphosphorylation of AIB1 occurs at mitosis exit. (A) Floating MCF7 cells arrested at mitosis with nocodazole were washed and liberated to progress through mitosis for the times indicated, in the presence or absence of 20 µM MG132 and 10 µg/ml cycloheximide (CHX). Western blot analysis was performed for AIB1 to monitor its phosphorylation status. Cyclin B1, protein phosphatase 1 (PP1), and the inhibitory phosphorylation of PP1 (P-PP1) were also assessed. β-actin was used as a loading control. (B) Flow cytometry analysis of MCF-7 cells treated as above was used to confirm cell-cycle progression as determined by DNA content.

To investigate the turnover of AIB1 during mitosis rounded up HeLa cells were collected after nocodazole treatment and allowed to recover in the presence of cycloheximide to block *de novo* protein synthesis. Based on flow cytometry analysis, cells treated with cycloheximide were able to complete mitosis, although no cyclin D1 was synthesized (Figure 5D). Under these conditions, the AIB1 band shift was barely detectable after 5 hours (Figure 5A, right panels). Furthermore, treatment with the proteasome inhibitor MG132 did not promote the accumulation of AIB1. These results demonstrate that phosphorylated AIB1 is not degraded by the proteasome and strengthen the argument that it is dephosphorylated at exit from M phase. Similar results were obtained with MCF-7 cells, although the dephosphorylation of AIB1 occured earlier in this cell line (Figure 6).

### AIB1 phosphatase is partially resistant to okadaic acid but inhibited by calyculin A

**Figure 7:**
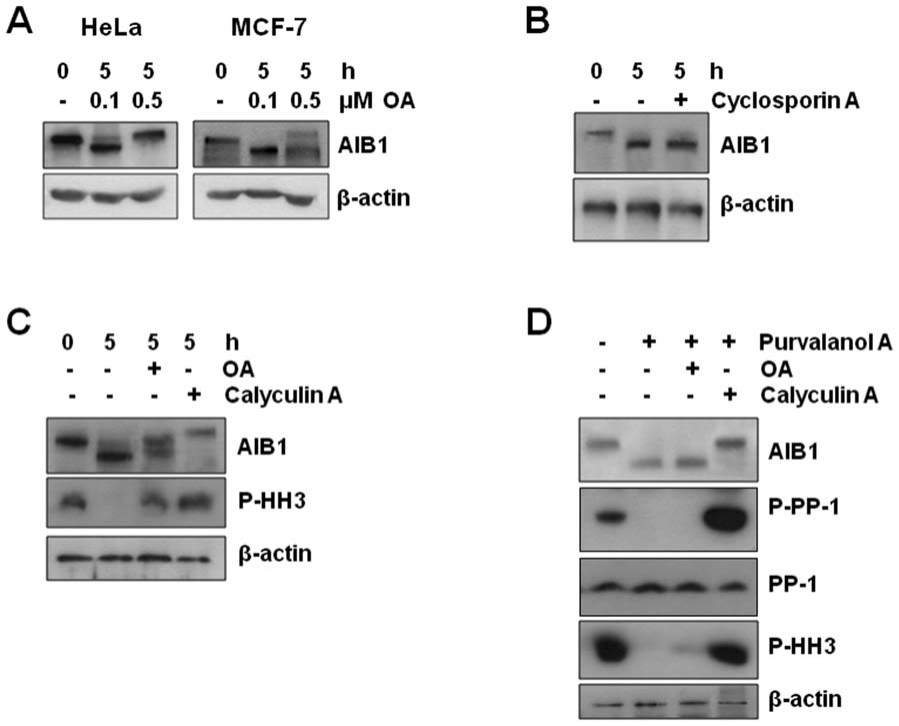
The dephosphorylation of AIB1 at the exit from mitosis is sensitive to okadaic acid and caliculin A inhibitors. (A) Mitotic HeLa and MCF-7 cells were allowed to progress through mitosis for the indicated times, in the presence or absence of the indicated concentrations of okadaic acid (OA). Cell lysates were analyzed subsequently by western blotting with AIB1 antibodies to detect the mitotic specific band and β-actin as loading control. (B) Western blot analysis of mitotic HeLa cells allowed to progress through mitosis for the indicated times, in the presence or absence of 5 µM cyclosporin A. (C) Rounded up HeLa cells arrested in mitosis as in (A) were allowed to progress through cell cycle in the absence (−) or presence (+) of 0.2 µM okadaic acid (OA) or 20 nM caliculin A for 5 hours. Cell lysates were analyzed by western blotting as indicated. (D) Western blot analysis of AIB1 phosphorylation status and other indicated molecular species from nocodazole-arrested HeLa cells further treated (+) with 10 µM purvalanol A, 0.2 µM okadaic acid (OA) or 20 nM caliculin A.

Okadaic acid is a specific inhibitor for phosphatases PP1 and PP2A. However, PP2A is much more sensitive to okadaic acid than PP1. To distinguish between the activities of these phosphatases, cell cultures were synchronized at mitosis and then were allowed to complete mitosis in the presence or absence of okadaic acid. The concentration was adjusted to inhibit PP2A alone (0.1 µM) or both PP1 and PP2A (0.5 µM). Okadaic acid blocked the dephosphorylation of AIB1 in HeLa cells in a dose-dependent manner (Figure 7A, left panels), suggesting that indeed PP1 dephosphorylates AIB1 at the exit of mitosis. In MCF7 cells, okadaic acid has been reported to more potently inhibit PP2A than PP1 [24]. Consistent with this, treatment with okadaic acid was much less efficient at blocking dephosphorylation of AIB1 in MCF-7 cells than in HeLa cells (Figure 7A, compare left and right panels). The PP2B specific inhibitor cyclosporine A had no effect on the dephosphorylation of AIB1 in HeLa cells (Figure 7B).

Calyculin A is a more effective inhibitor of both PP1 and PP2A than okadaic acid. Hence, we then compared these inhibitors in nocodazole-arrested HeLa cells. The dephosphorylation of AIB1 was only partially inhibited in the presence of okadaic acid (Figure 7C, third lane) whereas calyculin A completely blocked AIB1 dephosphorylation. One plausible interpretation of these results is that inhibition of the phosphatase prevents the inactivation of Cdk1, thereby maintaining AIB1 in a phosphorylated state. To evaluate this hypothesis, we further tested phosphatase inhibitors in the presence of purvalanol A. Similar to the results of Figure 4, treatment of nocodazole-arrested cells with the Cdk inhibitor purvalanol A promoted the dephosphorylation of AIB1 and the reactivation (absence of phosphorylation at threonine 320) of PP1 (Figure 7D). Treatment with 0.2 µM okadaic acid in the presence of purvalanol A did not prevent AIB1 dephosphorylation and PP1 reactivation. However, treatment with calyculin A blocked completely the dephosphorylation of AIB1 and increased PP1 phosphorylation/inactivation (Figure 7D). Collectively, these results demonstrate that AIB1 is dephosphorylated at the exit from mitosis, most likely by PP1.

### Cyclin B but not cyclin A participates in the Cdk- mediated phosphorylation of AIB1

**Figure 8:**
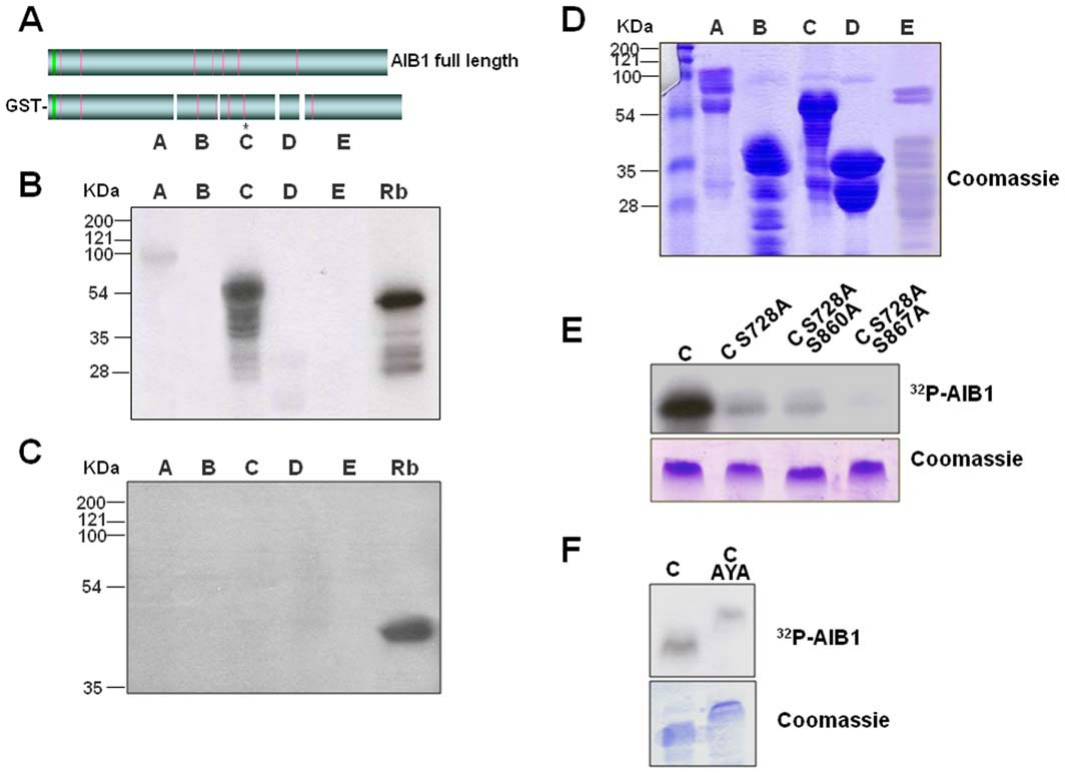
AIB1 is phosphorylated by Cdk1/cyclin B but not Cdk1/Cyclin A. (A) Representative scheme of full length AIB1 and GST fusion fragments generated and named A through E. (B) Qualitative analysis of AIB1 fragments subjected to *in vitro* phosphorylation with active Cdk1/cyclin B. Reactions were resolved by SDS-PAGE and gel, dried on Whatman paper and exposed for autoradiography. Fragment containing amino acids 792–928 of the Retinoblastoma protein (Rb) was also expressed as a GST fussion protein and 1 µg was used in a parallel reaction as a positive control. (C) Same fragments as in (B) were also incubated with 100 ng of active Cdk1/Cyclin A2 (Cell Signaling). (D) Coomassie staining of the different AIB1 fragments fused to GST. Based in the cualitative amounts of proteins stained, double input of fragment E was used in the radioactive reactions. (E) Autoradiography (upper panel) of AIB1 fragment C (aminoacids 693–933) and fragments harbouring point substitutions serine 728 to alanine (S728A), serine 860 to alanine (S860A) and serine 867 to alanine (S867A), subjected to phosphorylation by complex Cdk1/cyclin B1 in the presence of [γ-^32^P]ATP, 1 hour at 30°C. Bottom panel represents a Coomassie staining of the fragments used in the upper kinase reactions. (F) Autoradiography exposure (upper panel) and coomassie staining (lower panel) of AIB1 fragment C and a mutant (C AYA) in which amino acids RYL localized at +11 from serine 728 are mutated to AYA. Fragments were incubated with active Cdk1/cyclin B1 for 1 hour at 30°C in the presence of [γ-^32^P]ATP. Reactions were resolved by SDS-PAGE and gel, dried on Whatman paper and exposed for autoradiography. Fragment containing amino acids 792–928 of the Retinoblastoma protein (Rb) was also expressed as a GST fusion protein and used in a parallel reaction as a positive control.

To identify the site(s) targeted by Cdk1, we generated various AIB1 fragments (named A through E) that span the full-length sequence (Figure 8A). Fragments were expressed in bacteria (Figure 8D) and subjected to *in vitro* kinase assays with complexes of cyclin B1/Cdk1 (Figure 8B) or cyclin A2/Cdk1 (Figure 8C). Fragment C was highly phosphorylated by cyclin B1/Cdk1 but not by cyclinA/Cdk1. The amino-terminal fragment A was weakly phosphorylated by this complex but none of the other AIB1 fragments were radiolabeled upon incubation with cyclin A1/Cdk1 (Figure 8C), further supporting the notion that AIB1 is phosphorylated by Cdk1 just at the entry of M phase.

Interestingly, Fragment C contains a perfect consensus sequence for Cdk phosphorylation at S728. Additionally, eleven amino acids ahead of S728 is a cyclin binding motif (RXL), constituting a bipartite recognition motif for cyclin-dependent kinases [25]. To test whether the S728 serves as a substrate for cyclin B1/Cdk1, this site was mutated to alanine. The phosphorylation of the mutated Fragment C by Cdk1 was significantly reduced compared to the wild-type fragment (Figure 8E, compare C *vs.* C S728A). We also generated other point mutations and found that S867A combined with S728A completely abolished the Cdk1-mediated phosphorylation (Figure 8E, right lane). Moreover, mutation of RYL cyclin binding domain to AYA reduced by 50% the incorporation of radioactivity (Figure 8F), suggesting that it is required for complete phosphorylation of AIB1 by Cdk1. Our data support the notion that serine 728 is the major site for in vitro phosphorylation of AIB1, although we cannot exclude other sites that were not detected due such factors as the mis-folding of the recombinant protein fragments.

### Phosphorylated AIB1 is excluded from mitotic chromatin

**Figure 9:**
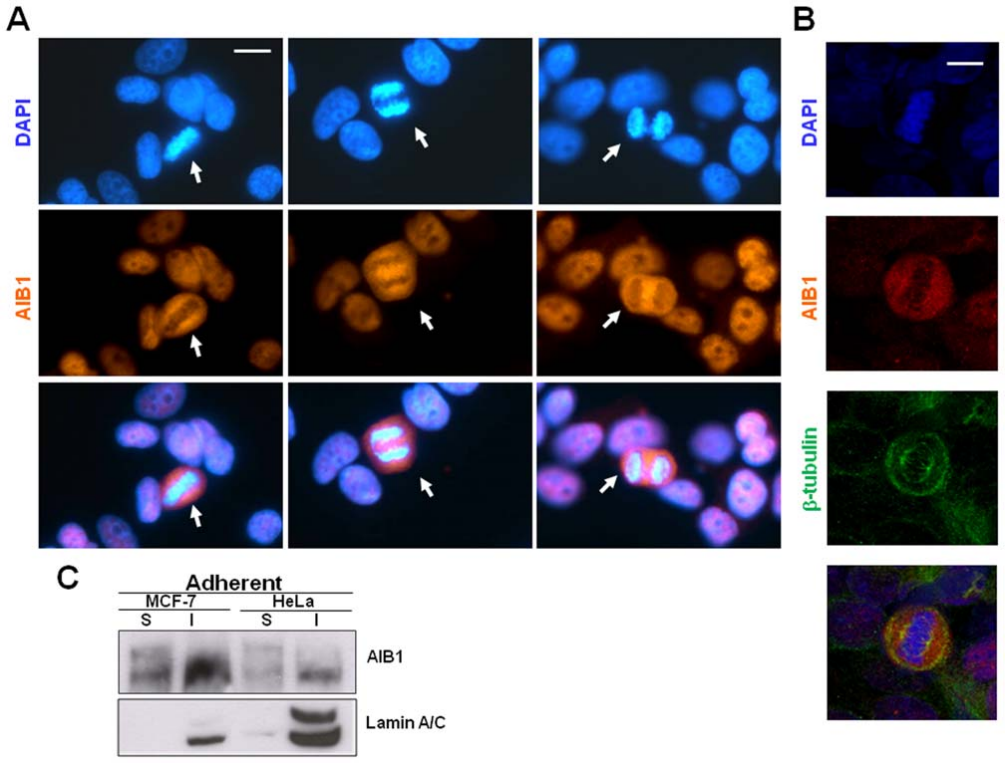
The band-shifted AIB1 is associated with the soluble fraction of cell lysates and is excluded from mitotic chromatin. (A) Asynchonous culture of MCF-7 cells were used for an immunofluorescence assay with anti-AIB1 antibodies (red) and DAPI (blue) to stain the chromatin. Bottom panels represent merged images. Scale bar: 10 µm. Mitotic cells are indicated with an arrow demonstrate that AIB1 is excluded from chromatin. (B) Confocal microscopy analysis of same cells as in (A) stained with anti-AIB1 (Alexa Fluor 488) and anti-β-tubulin (Alexa Fluor 633) to reveal the mitotic spindle. (C) MCF-7 and HeLa cells were arrested at mitosis with nocodazole and adherent cells were further subjected to cellular fractionation based on the treatment with two subsequent lysis buffers. The first buffer with low ionic strength completely solubilized the phosphorylated AIB1 (upper band, lane S). The second buffer containing high ionic strength extracted only the lower AIB1 band (lane I), together with the nuclear proteins lamin A/C, revealing non cross-contamination with the soluble fraction S.

**Figure 10:**
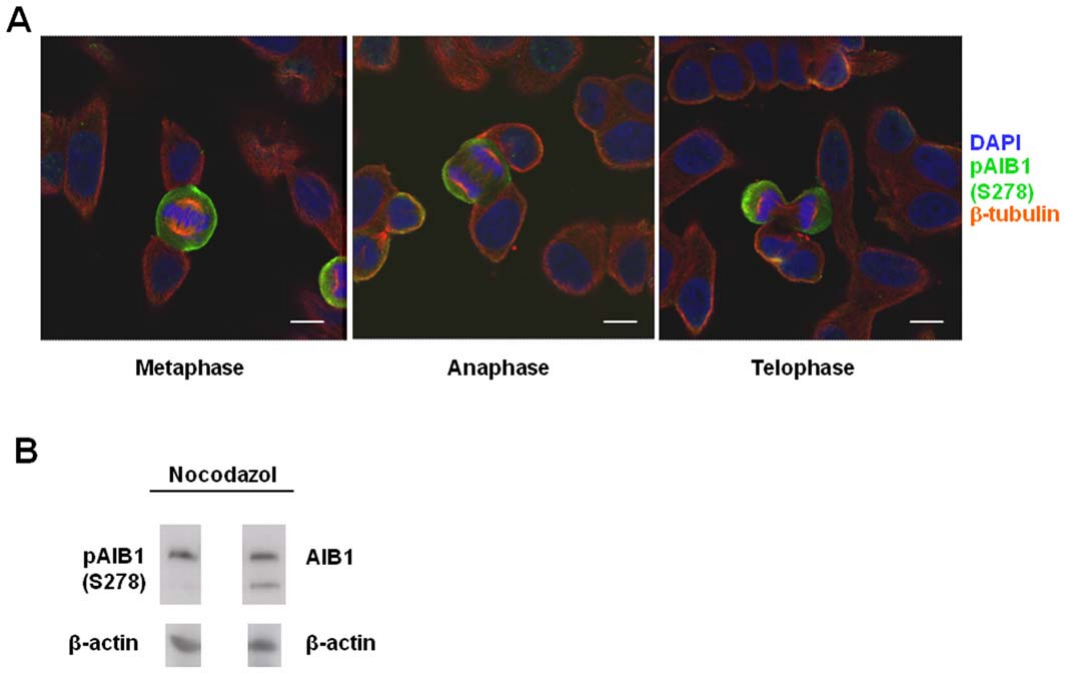
Phospho-specific antibodies (pAIB1) confirm that AIB1 is phosphorylated on Ser728 exclusively during mitosis. (A) Confocal microscopy analysis of an asynchonous culture of MCF-7 cells stained with DAPI (blue), anti-pAIB1(S728) (Alexa Fluor 488) and anti-β-tubulin (Alexa Fluor 633) to reveal the mitotic spindle. Scale bar: 10 µm. Only mitotic cells stain positively with pAIB1(S728) antibodies and suggest that pAIB1(S728) is excluded from chromatin. (B) Western blot analysis of MCF-7 cells arrested at mitosis with nocodazole. Cell lysates were probed with the indicated antibodies, revealing that anti-pAIB1(S728) antibodies detect exclusively the slower electrophoretic mobility band.

We next investigated whether the phosphorylation state of AIB1 might modulate its sub-cellular localization. Proliferating MCF-7 cells were stained with anti-AIB1 antibodies and mitotic cells were identified by DAPI or anti-tubulin staining. Our results reveal that AIB1 is excluded from chromatin in mitotic cells (Figure 9, A and B), suggesting that phosphorylation of this molecule may regulate its access to chromatin. To investigate the relation between Cdk1-mediated phosphorylation of AIB1 and its exclusion from chromatin, cells that adhered after nocodazole-treatment were lysed to obtain soluble (cytosol and nucleoplasm) and insoluble (enriched with proteins bound to chromatin and nuclear matrix) fractions [26]. Adherent cells contain phosphorylated and unphosphorylated AIB1 (Figure 1C, second lane) and thus, constitute the adequate cellular population to study differences between both AIB1 species. Interestingly, the mobility of AIB1 was retarded only in the soluble fraction (Figure 9C), whereas the band representing dephosphorylated AIB1 band was enriched in the insoluble fraction (Figure 9C, compare S and I lanes). These findings further support our observation that phosphorylation of AIB1 facilitates its exclusion from chromatin. We also generated phospho-specific antibodies directed against phosphorylated AIB1 at Ser728. These antibodies stained exclusively mitotic cells where AIB1 was localized to the periphery of the cell, suggesting its exclusion from chromatin during mitosis (Figure 10A). These results further suggest a correlation between the phosphorylation of AIB1 at Ser728 and its sub-cellular redistribution during mitosis. Consistent with this, western blot analysis with these phospho-specific antibodies detected only the retarded electrophoretic mobility band characteristic of AIB1 during mitosis (Figure 10B).

**Figure 11:**
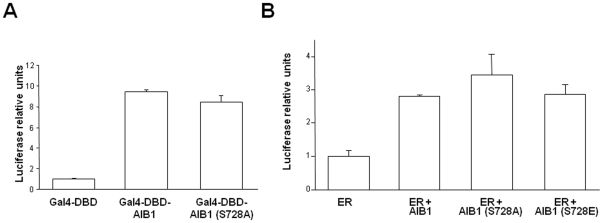
AIB1 phosphorylation at Serine 728 regulates its coactivation capacity. (A) To measure AIB1-dependent transcriptional activity, COS-1 cells were transfected with 500 ng of reporter gene (5×UAS-luciferase), 20 ng of tk-lacZ and 25 ng of empty vector pCMX-Gal4-DBD (control, C) or 46.5 ng of pCMX-Gal4-DBD-AIB1 wild type or mutant. 30 hours after the transfection cells were arrested at mitosis with nocodazole (150 ng/ml) for 17 hours. Finally, cells were lysed with passive lysis buffer (Promega) and tested for luciferase activity and normalized against β-gal activities in a luminometer (Berthold Detection Systems). Results are presented as relative units to the control cells as mean values ± S.E. (B) To measure AIB1-dependent coactivation, COS-1 cells were transfected with the luciferase reporter ERE2-tk-luciferasa and vectors expressing ERα, AIB1 or the mutated AIB1 (S728A) or (S728E). Cells were arrested at mitosis similarly to (A) and treated with 50 nM 17-β-estraxdiol during the last 5 hours.

To assess the effects of phosphorylation on the transcriptional activity of AIB1, we fused full-length AIB1 to the DNA binding domain of Gal4 (Gal4-DBD) and co-transfected it with the luciferase reporter driven by the Gal4 responsive element. COS1 cells were treated with nocodazole prior to analysis. No significant differences were detected between AIB1 with the mutation at S728 and wild-type AIB1 (Figure 11A). Alternatively, phosphorylation could alter the capacity of AIB1 to bind other transcriptional factors without affecting its capacity to activate the transcriptional machinery. To test this alternate hypothesis, we used an estrogen receptor alpha (ERα)-dependent co-activation assay. Neither of the AIB1 mutants (S728A or S728E) displayed notable differences of coactivation capacity when compared to wild-type AIB1. These results suggest that the phosphorylation of AIB1 during mitosis does not modulate its transcriptional capacity and ability to coactivate ERα.

## Discussion

Phosphorylation of proteins during mitosis and their dephosphorylation at the exit from mitosis require precise regulation to maintain the integrity of the cell-cycle. The function of the transcriptional coactivator AIB1 has been studied extensively in the progression of G1 to S but little is known about its role in M phase. AIB1 is the target of various kinases that regulate both its function and its turnover. Our results demonstrate that AIB1 also serves as a substrate of Cdk1/cyclin B just at the entrance of mitosis, thus providing a new mechanism for regulating AIB1 during the cell cycle. We found that phosphorylation of AIB1 occurs solely in mitotic cells and that this molecule remains phosphorylated throughout mitosis. Consistent with this, Cdk1 associated with cyclin B not cyclin A was able to phosphorylate AIB1 *in vitro*, suggesting that this phosphorylation event does not occur at G2 but rather exclusively at M phase, when the cyclin B/Cdk1 complex is active in dividing cells.

Mitogenic stimuli that promote cell-cycle progression through G1/S trigger the activation of signaling kinases such as ERKs and AKT. Phosphorylation of AIB1 by ERKs at multiple residues results in the recruitment of p300 and enhances its coactivation capacity [21]. On the other hand, PI3K/AKT can stabilize AIB1 and prevent its degradation by the proteasome [7]. GSK3 can also phosphorylate AIB1 at S505, coupling AIB1 to mono-ubiquitylation and further poly-ubiquitylation at K723 and K786, resulting in its recognition by the proteasome [20]. Ubiquitylation at K1194 has also been detected, but it does not induce proteasomal degradation, although it is required for a full coactivation by the proteasome [7]. Thus, activation of the ERK and PI3K/AKT pathways enhance AIB1 activity and stability. To investigate whether the phosphorylation of AIB1 during M is also associated with ubiquitylation, we used three different experimental strategies (Figure 2). However, we were unable to demonstrate that mitotic AIB1 is ubiquitilated, suggesting that phosphorylation of AIB1 during mitosis does not result in ubiquitin-proteasome degradation or ubiquitin-dependent trafficking. Moreover, AIB1 protein levels remain constant throughout mitosis (Figure 5), providing additional evidence that the phosphorylation at the onset of M is not associated with degradation but may rather regulate the activity of AIB1. Treatment of phosphorylated AIB1 with protein phosphatase alone completed restored the doublet to a single band, suggesting that phosphorylation was the only post-translational modification that occurs to AIB1 at mitosis. A previous study has shown that sumoylation coordinated with phosphorylation regulates the transcriptional activity of AIB1 [27]. However, our attempts to detect sumo in complexes of phosphorylated AIB1 with specific antibodies produced negative results (data not shown).

We have identified two serines (728 and 867) which were phosphorylated by Cdk1 in an *in vitro* kinase assay. Ser728 is potently phosphorylated by Cdk1 and is within the motif (S/T)PX(R/K) (where X is any amino acid) found in most Cdk substrates. Both sites have recently been reported to be phosphorylated using a high throughput proteomics analysis [28]. However, the data in this study suggest that they are predominantly phosphorylated at G1. In contrast, our results demonstrate that phosphorylation at those sites occurs only by Cdk1/cyclinB. In addition, we show that AIB1 is dephosphorylated at the exit from mitosis. Hence, our data suggest strongly that phosphorylation occurs at these sites during mitosis but not during G1. Sequence analysis revealed a cyclin binding motif localized 11 aminoacids downstream of S728. Mutation of this motif reduced but did not attenuate completely the phosphorylation at S728 by the cyclinB/cdk1 complex. These results suggest that the cyclin binding motif is relevant but not essential for AIB1 phosphorylation by Cdk1.

During interphase, transcription factors and related proteins are frequently redistributed within the nucleus of cells. Many of these proteins (such as RNA polymerase II) are displaced from chromatin during mitosis, whereas others like TFIID and TFIIB can remain associated with active gene promoters during mitosis [29]. Immunofluorescence staining revealed that in mitotic cells AIB1 is excluded completely from the condensed chromatin. Interestingly, phosphorylation of the transcriptional coactivator BRG1 during mitosis correlates with decreased affinity for nuclear structure but does not disrupt the association with SNF5 [26]. Similarly, we found that phosphorylated AIB1 associated with mitosis is exclusively detected in the soluble fraction that lacks chromatin-bound proteins. In our experiments to test whether phosphorylation of AIB1 during mitosis modulates its transcriptional activity, we used transient transfection of plasmids harboring the luciferase reporter gene under the control of either Gal4-DBD or ER responsive elements. These results demonstrate that, in contrast to its exclusion from native chromatin during M phase of dividing cells, AIB1 and other factors of the basal transcriptional machinery are recruited to the luciferase promoter to transcribe the luciferase gene during mitosis. One plausible explanation for these results is that these plasmids and their minimal promoters are not subjected to the same epigenetic complexities as endogenous promoters of the chromatin. Point mutations at Ser728 of AIB1 did not alter the endogenous transcriptional activity or the capacity to coactivate ERα. These results reveal that phosphorylation of AIB1 on S728 by Cdk1 does not regulate its activity or association with transcription factors. However, it remains possible that phosphorylation at additional sites would induce changes in the transcriptional capacity of AIB1. Nevertheless, phosphorylation by Cdk1 promotes exclusion from the chromatin and therefore, physically restricts AIB1-dependent transcriptional activation during mitosis. Based on this model, dephosphorylation of AIB1 at exit from M would restore its capacity to coactivate the transcription of genes implicated in progression through G1. PP1 has previously been reported to dephosphorylate AIB1 at residues S101 and S102 [30], thus preventing its ubiquitylation and degradation. However, our findings demonstrate that dephosphorylation of AIB1 by PP1 at the exit from mitosis does not influence protein stability but rather facilitates subsequent re-entry of the cell cycle. Thus, dephosphorylation of AIB1 may represent a novel biological target for therapeutic interventions aimed at improving the outcome of current treatments of AIB1-dependent cancers.

## Materials and Methods

### Cell culture, cell transfection and cell-cycle synchronization

HeLa, MCF-7 and COS-1 cells (ATCC) were maintained in DMEM – 10% FBS (Invitrogen). Sf9 cells (Invitrogen) were cultured in SF-900 II medium (Invitrogen). Transfections in COS-1 cells were performed with FuGene 6 (Roche) according to manufacturer instructions. For synchronization, cells were deprived of serum for 24 h and released in the presence of one of the following inhibitors: 16 h, 5 µM cyclosporin to arrest at G0/G1; 24 h with 100 nM wortmannin for late G1, 24 h with 400 µM mimosine for G1/S, 48 h with 5 mM hydroxyurea for S-phase, 24 h with 10 µM etoposide for G2/M and for 17 h with 150 ng/ml nocodazole for M-phase (prometaphase) followed by shake-off to collect mitotic cells. All inhibitors were purchased from Sigma. Mitotic cells were washed and released for the indicated times in the presence or absence of 10 µg/ml cycloheximide (Sigma), 20 µM MG132 (Calbiochem), 0.1–1 µM okadaic acid (Calbiochem) and 20 nM calyculin A (Calbiochem). Alternatively, mitotic cells were resuspended in nocodazole containing media supplemented with 20 µM PD98059 (Sigma) or 10 µM purvalanol A (Calbiochem) alone and/or okadaic acid or calyculin A for 2 h.

### Flow cytometry analysis

Cells were harvested by trypsin treatment, washed with PBS and resuspended in 70% ethanol to permit fixation and permeabilization at −20°C. Propidium iodide-stained cells (5 µg/ml; Sigma) were analyzed using a flow cytometer (Cytomics FC500; Beckam Coulter).

### Protein extraction and Western blotting

Cells were washed with ice-cold PBS and lysed with commercial lysis buffer (Cell Signaling) supplemented with protease cocktail inhibitors (Roche), 1 mmol/l NaF and up to 300 mM NaCl. His6-tagged proteins were lysed with 6 M guanidium-HCl, 0.1 M Na_2_HPO_4_/NaH_2_PO_4_ and 0.1 M Tris-HCl pH 8, sonicated for 30 s and centrifuged for 15 min at 16000×g. Cleared supernatant was diluted 1∶1 in PBS, and His6-tagged proteins were purified by Ni^2+^-NTA affinity chromatography (Qiagen) in the presence of 10 mM Imidazole (Merck), 1 mM AEBSF (Roche), 20 µM MG132, 10 mM N-etilmaleimide (Sigma) and 5 nM ubiquitin aldehyde (Santa Cruz Biotech) during 2 h at room temperature. Agarose beads were then washed once with buffer A (same as lysis buffer but with 10 mM imidazol), once with buffer B (8 M urea, 0.1 M Na_2_HPO_4_/NaH_2_PO_4_, 0.01 M Tris-HCl pH 8 and 10 mM imidazol), four times with decreasing concentrations of Triton X-100 (starting at 0.2%) in buffer C (8 M urea, 0.1 M Na_2_HPO_4_/NaH_2_PO_4_, 0.01 M Tris-HCl pH 6.3 and 10 mM imidazol). Finally, ubiquitylated proteins were eluted by boiling in Laemmli buffer.

Subcellular fractionation was done as previously reported [26] with the following adaptation: the insoluble pellet was extrated with lysis buffer.

Samples were resolved in 7.5% or 15% SDS-PAGE, blotted to Immobilon-P membranes (Millipore) and probed with the following antibodies: monoclonal anti-AIB1 (BD Biosciences), polyclonal anti-phospho-AKT Ser473, polyclonal anti-phospho-Cdc2 Tyr15, polyclonal anti-phospho-PP1 Thr320 and polyclonal anti-Cdc2 (Cell Signaling); monoclonal anti-phospho-ERK1/2 Tyr204, polyclonal anti-cyclin A, polyclonal anti-cyclin B1, monoclonal anti-cyclin D1, polyclonal anti-cyclin E, polyclonal anti-PP1, monoclonal anti-Lamin A/C from (Santa Cruz Biotech); monoclonal anti-phospho-histone H3 from Millipore; monoclonal anti-β-actin from Sigma and monoclonal anti-ubiquitin FK2 (Affinity Bioreagents). Phospho-specific anti-pAIB1(S728) were raised in rabbits against the peptide KQEQL(pS)PKKKENNA. Sera from the immunized rabbits were cleared with the unphosphorylated peptide.

### Immunoprecipitation

Pre-cleared whole-cell lysates (1 mg) were immunoprecipitated with polyclonal AIB1 antibodies (generated in our laboratory) during 4 h at 4°C. Protein A-agarose was added to immunocomplexes and incubated for 2 more hours. Immunocomplexes were washed three times with lysis buffer and solubilized by boiling in Laemmli buffer. Half of the immunoprecipitation was analyzed by western blot. Preimmune serum was used as negative control.

### DNA constructs and site directed mutagenesis

Plasmid constructs pGEX4T-1-AIB1(1–555), pGEX4T-3-AIB1(693–933), pGEX4T-3-AIB1(934–1031) and pGEX4T-1-AIB1(1032–1424) were generated by subcloning cleaved fragments from pcDNA3.1+AIB1 with SspI and EcoRI. Construct pGEX4T-1-AIB1(556–693) was generated by PCR to add EcoRI-SalI sites. Point mutations S728A, S728E, S860A, S867A and R739A/L741A were generated using QuickChange site-directed mutagenesis kit (Stratagene). Mutations were verified by sequencing.

*Kinase assay-* Recombinant full-length AIB1 protein produced in Sf9 cells (1 µg) or GST-AIB1 fragments attached to the sepharose beads (15 µl) were subjected to *in vitro* phosphorylation in a final volumen of 30 µl with kinase buffer, 10 units of Cdk1-cyclin B1 complex (Cell Signaling), 200 µM ATP and 5 µCi [γ-^32^P]-ATP (Perkin Elmer). Reactions were incubated for 1 h at 30°C, analyzed by SDS-PAGE and exposed for autoradiography. 1 µg of Histone H1 (Sigma) and 1 µg of GST-Retinoblastoma (aminoacids 792–928) were used as positive controls. *In vitro* phosphorylation by Cdk1-cyclin A2 complex (Cell Signaling) was performed similarly as above, except that 100 ng of Cdk1-cyclin A2 complex were used per reaction.
